# Left coronary ostial isolation in a young boy caused by a dysplastic aortic valve: a case report

**DOI:** 10.1093/ehjcr/ytaf012

**Published:** 2025-01-20

**Authors:** Nicholas Fitzgerald, Matthew Liava’a, Ganesh Gnanappa, Julian Ayer

**Affiliations:** The Heart Centre for Children, The Children’s Hospital at Westmead, Corner of Hawkesbury Road and Hainsworth Street, Westmead, New South Wales 2145, Australia; The University of Sydney Children's Hospital at Westmean Clinical School, Corner of Hawkesbury Road and Hainsworth Street, Westmead, New South Wales 2145, Australia; The Heart Centre for Children, The Children’s Hospital at Westmead, Corner of Hawkesbury Road and Hainsworth Street, Westmead, New South Wales 2145, Australia; The Heart Centre for Children, The Children’s Hospital at Westmead, Corner of Hawkesbury Road and Hainsworth Street, Westmead, New South Wales 2145, Australia; The Heart Centre for Children, The Children’s Hospital at Westmead, Corner of Hawkesbury Road and Hainsworth Street, Westmead, New South Wales 2145, Australia; Discipline of Child and Adolescent Health, The Children’s Hospital at Westmead Clinical School, Corner of Hawkesbury Road and Hainsworth Street, Westmead, New South Wales 2145, Australia

**Keywords:** Case report, Chest pain, Myocardial ischaemia, Dysplastic aortic valve, Isolation of the left coronary ostium, Paediatric Surgery

## Abstract

**Background:**

Ischaemic cardiac chest pain and coronary artery abnormalities are uncommon in children. The long-term implications of missed or delayed diagnosis are myocardial ischaemia and risk of sudden cardiac death. Improvement in non-invasive imaging has made diagnosis and surgical planning possible with multi-modal imaging.

**Case summary:**

A 12-year-old boy with ischaemic chest pain caused by isolation of the left coronary ostium in the context of a dysplastic aortic valve. There was a delay to formal diagnosis. Surgical aortic valve repair resulted in complete resolution of symptoms. Ethics approved (SCHN: CCR2023/5).

**Discussion:**

Isolation of the left coronary ostium caused by a dysplastic aortic valve (without supravalvar stenosis) is an example of a rare cause of ischaemic chest pain in children. To our knowledge, only 10 paediatric case reports are published in English. In reported cases, presenting features varied from poor feeding and a murmur in infants to chest pain, syncope, or cardiac arrest in adolescents. Historically, angiography during a cardiac catheter procedure was required for diagnosis; however, improvements in non-invasive imaging techniques have resulted in the diagnosis being possible on echocardiography (supported by computed tomography angiography or cardiac magnetic resonance imaging).

**Learning points**

- A high index of suspicion is required to diagnose coronary ostial anomalies in patients presenting with chest pain and a dysplastic aortic valve.
- Diagnosis and surgical planning are possible on non-invasive, multi-modal imaging.
- Awareness and timely diagnosis are essential to prevent irreversible ischaemic myocardial damage or sudden cardiac death.

## Introduction

Ischaemic cardiac chest pain is uncommon in children.^1^ Coronary ostial obstruction is a known association of supravalvar aortic stenosis but is rare with an isolated dysplastic aortic valve.^2^ We report a case of ischaemic chest pain due to isolation of the left coronary ostium caused by a dysplastic aortic valve in a 12-year-old boy. The long-term implications of missed or delayed diagnosis are myocardial ischaemia and risk of sudden cardiac death. Improvement in non-invasive imaging has made diagnosis and surgical planning possible with multi-modal imaging.^3^

## Summary figure

**Figure:**
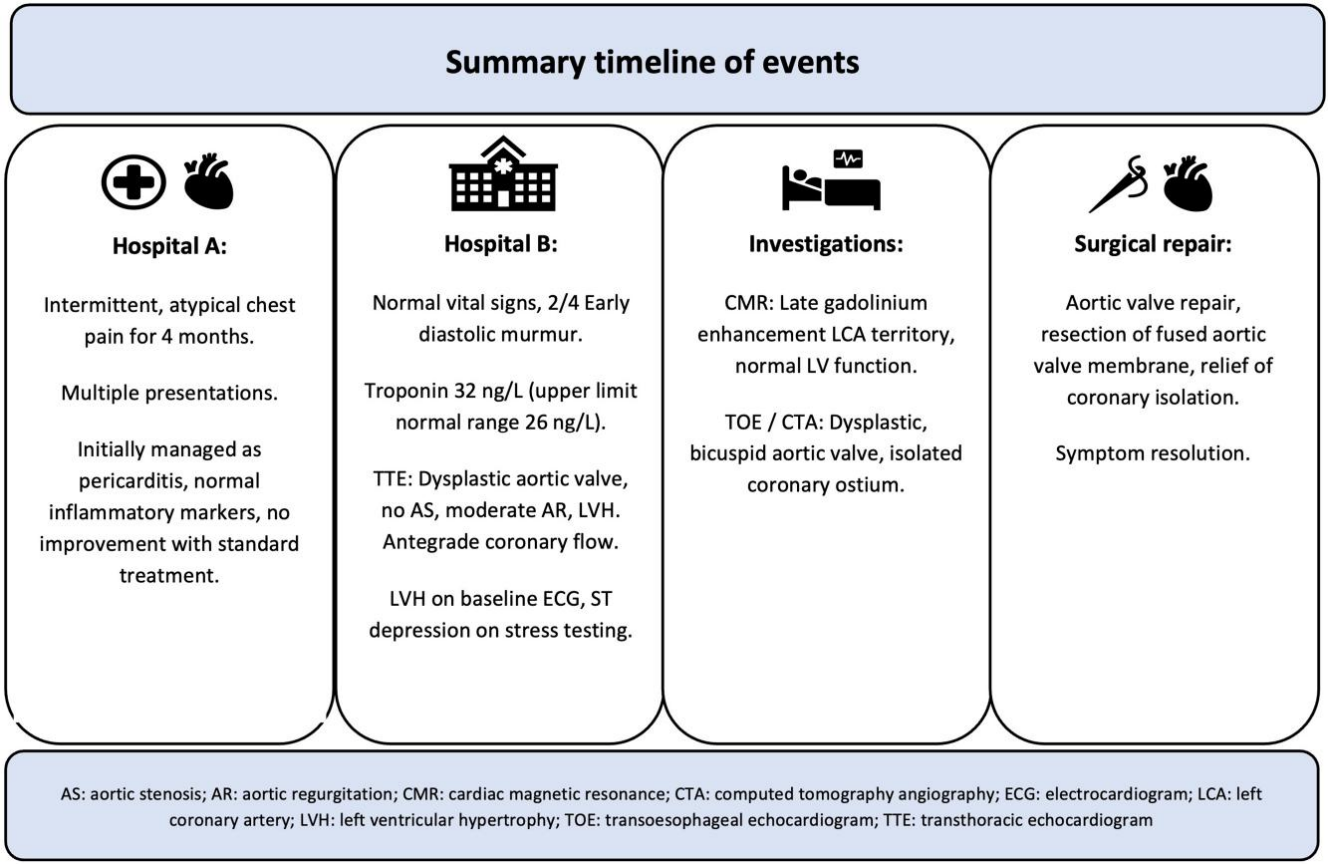


## Case presentation

A previously well 12-year-old boy was referred to our tertiary paediatric cardiology service with a four-month history of episodic and progressive severe, atypical, left-sided chest pain which caused significant functional disturbance and analgesia requirement. It was reported at rest and with exertion. He would assume strange back arching postures for symptom relief. No other cardiac symptoms were reported. An echocardiogram performed at the referring centre demonstrated a dysplastic aortic valve with mild aortic regurgitation (AR) and left ventricular (LV) hypertrophy. Serial troponin-I testing in the week prior to referral to our unit revealed only one elevated result of 32 ng/L (upper limit normal 26 ng/L). There was no family history of cardiac disease. Previous recent local hospital presentations were managed as presumed pericarditis, though there was no improvement with anti-inflammatory treatment. The differential included cardiac disease, musculoskeletal pain, gastro-oesophageal reflux and due to the non-specific nature of the chest pain and the significant functional impact (withdrawal from activity, low mood, school absence), a non-organic cause was also considered. Pharmacological treatment (simple and opioid analgesia as required, a proton pump inhibitor for reflux and tricyclic anti-depressant) as well as non-pharmacological strategies were commenced with mild interval improvement in the symptoms.

On our initial assessment, vital signs were normal (HR 80 beats/minute, BP 115/60 mmHg, oxygen saturation 99% in air, afebrile). Peripheral pulses and heart sounds were normal with a 2/4 early diastolic murmur. There were no signs of heart failure or peripheral stigmata of infective endocarditis.

Laboratory testing demonstrated a normal blood count, liver/renal function, and markers of inflammation. The chest X-ray was normal. The electrocardiogram demonstrated a sinus rhythm with LV hypertrophy. No arrhythmia was documented during monitoring. A transthoracic echocardiogram demonstrated a dysplastic, functionally bicuspid aortic valve with a hypoplastic, restricted left coronary cusp (LCC). There was mild-moderate AR and no aortic stenosis (supplementary material online, Videos S1 and S2). The coronary origins were assessed as appearing normal with antegrade colour doppler flow demonstrated and aortic arch flow was unobstructed. There was mild global LV hypertrophy with normal systolic function.

**Figure 1:**
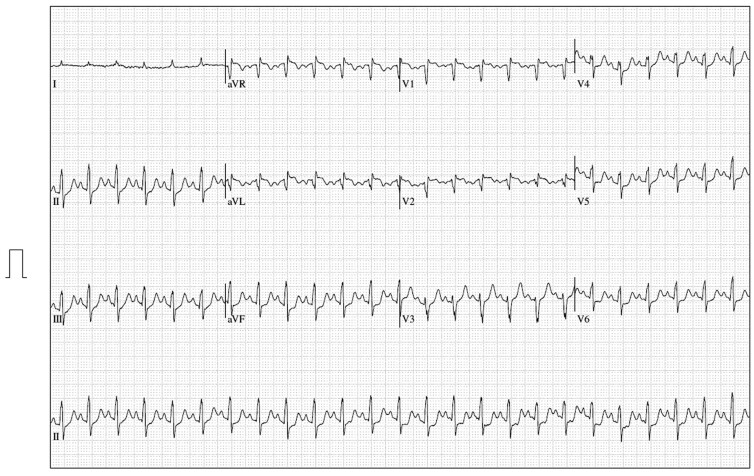
Twelve-lead ECG immediately following the treadmill exercise stress test demonstrating infero-lateral ST segment depression.

A Standard Bruce Protocol treadmill exercise test was performed. Although pain was present at rest, the test was terminated due to worsening chest pain. There was mild infero-lateral ST segment depression (*Figure 1*). No regional wall motion abnormality was identified on stress echocardiogram after peak exercise.

**Figure 2:**
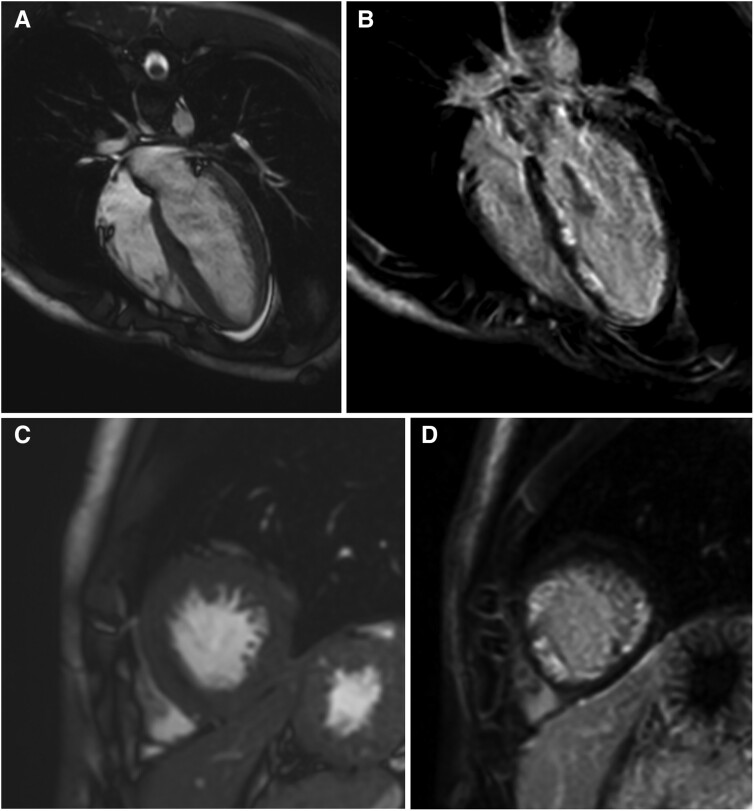
Cardiac magnetic resonance comparing cine and delayed gadolinium enhanced images in four chamber (*A* and *B*) and short axis views (*C* and *D*) demonstrating subendocardial infarct pattern in the left ventricle.

Given the presence of LV hypertrophy, a cardiac magnetic resonance imaging (MRI) was requested but deferred due to pain and an inability to comply with breath-holding and lying flat. When performed four months later, widespread subendocardial late gadolinium enhancement in the left anterior descending and left circumflex coronary artery territories was demonstrated (*Figure 2*). LV systolic function was normal.

**Figure 3:**
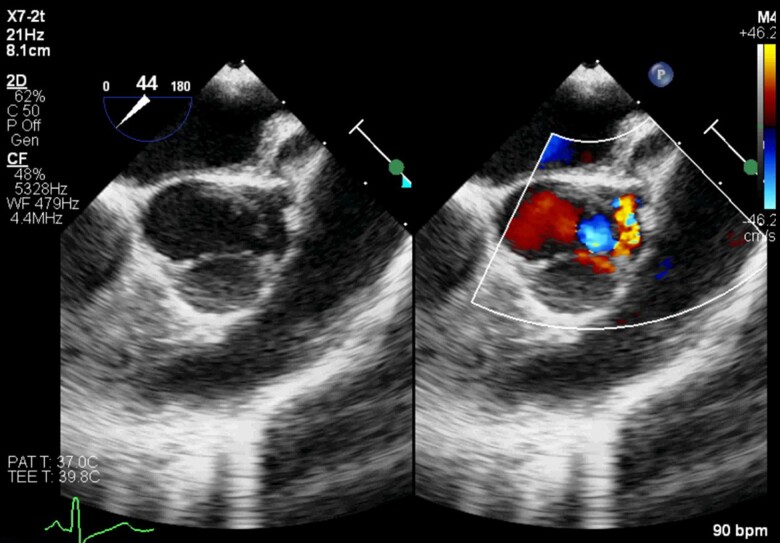
Transoesophageal echocardiogram with the aortic valve in short axis demonstrating the hypoplastic left coronary cusp (LCC), isolated coronary ostium, and obstructed coronary artery flow.

After the MRI, a transoesophageal echocardiogram demonstrated that the free margin of the LCC was adherent to the aortic wall, creating a pouch. The origin of the left coronary artery (LCA) was isolated as it arose from the pouch. Flow was noted into the pouch through an opening at the margin of a small fenestration in the cusp in late diastole, but with a colour flow pattern suggestive of obstruction (*Figure 3*, supplementary material online, Video S3). The right and non-coronary cusps were normal. There was mild aortic regurgitation from a centrally located jet.

**Figure 4:**
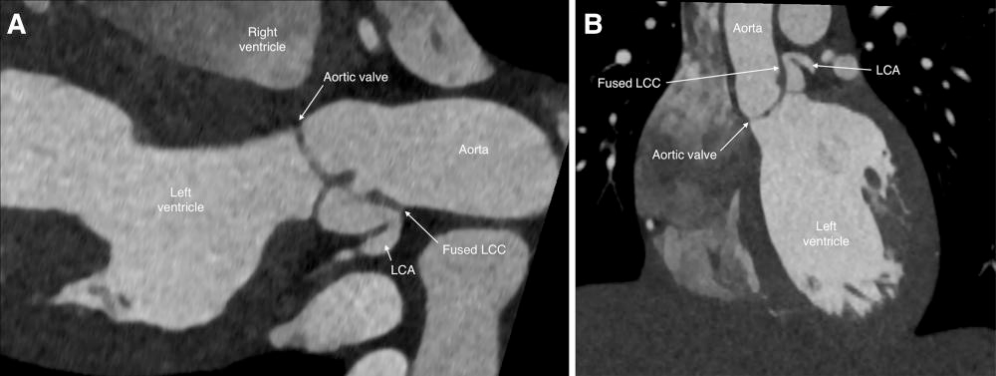
Long axis (*A*) and coronal (*B*) reconstructions from computed tomography angiogram demonstrating the fused left coronary cusp (LCC) and pathway to the left coronary artery (LCA).

A computed tomography (CT) angiogram confirmed a dysplastic, bicuspid aortic valve with a retracted LCC attached to the aortic wall above the LCA origin. Blood flow to the left coronary sinus entered through a small opening of the LCC at the inferior–anterior margin of the leaflet (*Figure 4*, supplementary material online, Video S4). The LCA arose from the isolated left sinus of Valsalva and its ostium was at the upper margin of the sinus with subsequent angulation of 70 degrees of the left main coronary artery. The right coronary artery origin and size were normal.

**Figure 5:**
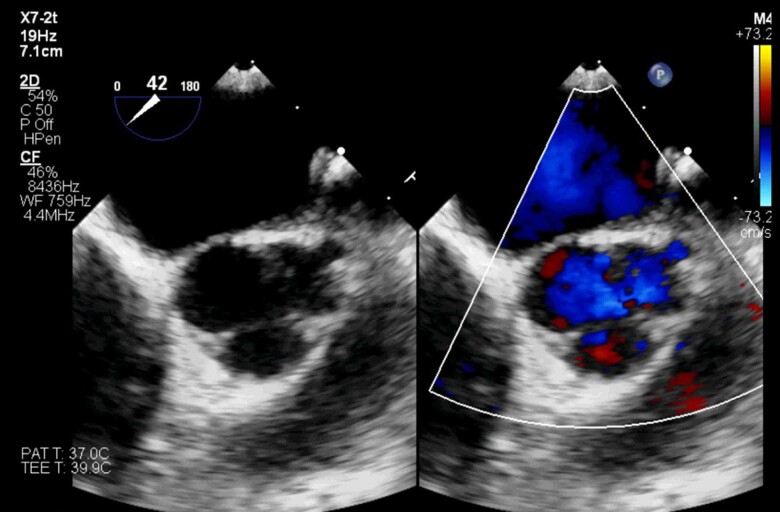
Transoesophageal echocardiogram demonstrating unobstructed left coronary artery flow post repair.

Surgical repair strategy was pursued in the setting of a symptomatic presentation with coronary ostial obstruction and resultant myocardial ischaemia. The dysplastic LCC had a thick membrane attached which was fused to the aortic wall and covered the left coronary ostium. There was a small opening to the left coronary sinus between the free edge of the LCC, aortic wall and free edge of the membrane (see Supplementary material online, *Figure S1*). The coronary cusp was separated from the aortic wall and the membrane was excised, leaving a freely mobile LCC. Residual tissue above the coronary ostium was resected, resulting in unobstructed flow into the LCA. Intra-operative transoesophageal echocardiogram demonstrated unobstructed flow to the left coronary ostium and trivial aortic regurgitation (*Figure 5*).

Post-operative recovery was uneventful with immediate relief of the chest pain. Aspirin was commenced for the aortic valve repair and Atenolol in the setting of the subendocardial infarct plus concern for ventricular arrhythmia risk. At six-week follow up, the patient remained pain free. An electrophysiology study undertaken at three months post-surgery to assess for inducible arrhythmia did not demonstrate inducible monomorphic ventricular tachycardia. In the case of a positive study, an ablation procedure or implantable cardioverter–defibrillator would have been considered. At 12 months post-surgery, the patient remained asymptomatic, an implanted loop-recorder had not revealed any arrhythmia, persisting extensive subendocardial late Gadolinium enhancement was present on a cardiac MRI and a CT angiogram demonstrated an unobstructed LCA.

## Discussion

Isolation of the left coronary ostium caused by a dysplastic aortic valve is an example of a rare cause of ischaemic chest pain in children. To our knowledge, only 10 paediatric case reports are published in English (see Supplementary material online, *Table S1*). We are not aware of a case where coronary isolation resulted from a discrete membrane arising from the coronary cusp. In reported cases of coronary isolation, the age of presentation varied from infancy, characterized by poor feeding and a murmur, to older children and adolescents presenting with chest pain, syncope or cardiac arrest.^4–8^ Historically, angiography during a cardiac catheter procedure was required for diagnosis, however improvements in non-invasive imaging techniques have resulted in the diagnosis being possible on echocardiography (supported by CT angiography or cardiac MRI).^3^ Management depends on the valve morphology and location of the coronary ostium. Most commonly, aortic valve repair or replacement was undertaken. A Ross–Konno procedure was performed in one infant and in another, coronary translocation and interposition grafting was required.^7,8^

## Conclusion

Isolation of the coronary ostium caused by a dysplastic aortic valve is a rare but potentially remediable cause of ischaemic chest pain. Awareness of this condition is essential to expedite diagnosis and potentially prevent ischaemic myocardial damage or sudden cardiac death. Coronary obstruction should be considered in the setting of recurrent chest pain with a dysplastic aortic valve, even if antegrade coronary flow is demonstrated on echocardiography.

## Supplementary Material

ytaf012_Supplementary_Data

## Data Availability

All relevant data are incorporated into the article and its online Supplementary material.

## Lead author biography

Nicholas Fitzgerald has just completed an Interventional Catheterisation Fellowship at The Hospital for Sick Children, Toronto (Canada). His core cardiology fellowship training was completed at The Children’s Hospital at Westmead, Sydney (Australia). He graduated from the University of New South Wales (2014) with an MBS/MD and from The University of Sydney (2017) with a Master of Medicine (Clinical Epidemiology).

## Supplementary material

**Consent:** The authors confirm that written consent was obtained for the publication of case details and related images/videos in line with the COPE best practice guidelines prior to submission and publication of this case report. Institutional ethics approval was obtained (SCHN: CCR2023/5).
