# Supplementary material for: Left coronary ostial isolation in a young boy caused by a dysplastic aortic valve: a case report
Source: Eur Heart J Case Rep. 2025 Jan 20;9(2):ytaf012. doi: 10.1093/ehjcr/ytaf012 (PMC11840340; doi:10.1093/ehjcr/ytaf012)
Supplement: ytaf012_Supplementary_Data [file ytaf012_supplementary_data.zip › Appendix Table 1_with reference list.docx]

**Appendix Table 1:**

| **Year** | **Age** | **Presenting symptom(s)** | **Imaging** | **Aortic valve** | **Valve function** | **Coronary ostial obstruction** | **Operation** |
| --- | --- | --- | --- | --- | --- | --- | --- |
| 1970^1^ | 16 years | Chest pain, syncope | CA | Tricuspid | Mild AS, moderate AR | Complete | AVR |
| 1981^2^ | 8 years | Chest pain | CA | Tricuspid | AS, mild AR | Partial | AVR, sub-aortic membrane resection |
| 1981^2^ | 9 years | Chest pain | CA | Tricuspid | Moderate-severe AR | Partial | AVR, coronary interposition graft |
| 1981^3^ | 16 years | Sudden death | Nil | Quadricuspid | N/A | Complete | Nil |
| 2005^4^ | 10 years | Chest pain | TTE, CA | Quadricuspid | N/A | Partial | Aortic valve replacement |
| 2010^5^ | 13 years | Chest pain, syncope | TTE, TOE | Tricuspid | Mild AS/AR | Partial | AVR |
| 2010^6^ | 2 years | Heart murmur | TTE, CTA, CA | Tricuspid | Moderate AS | Partial | Ross-Konno Surgery |
| 2017^7^ | 2 months | Poor feeding, failure to thrive | TTE, Catheter | Quadricuspid | Moderate AR | Partial | Translocation of coronary origin and autologous pericardial patch augmentation of LMCA |
| 2020^8^ | Antenatal | Cardiomegaly, LV dilated with EFE | TTE, CA, CTA, C-MRI | Tricuspid | Moderate AR | Partial | AVR |
| 2022^9^ | 6 weeks | Heart murmur, poor feeding, failure to thrive | TTE, CTA, CA | Bicuspid | Moderate AS, mild AR | Partial | AVR, homograft patch repair of supravalvar AS. |
| 2022^10^ | 2 months | Cardiac arrest | TTE, CTA | Quadricuspid | Mild AR | Partial | AVR |

Appendix Table 1: Summary of previously published case reports for coronary ostial obstruction in the paediatric population. AS: aortic stenosis, AR: aortic regurgitation, AVR: aortic valve repair, CA: catheter angiography, CTA: computed tomography angiography, EFE: endocardial fibroelastosis, LV: left ventricle, TTE: transthoracic echocardiogram, TOE: transoesophageal echocardiogram

**References**

1. Waxman B, Kong Y, Behar V, Sabiston D, Morris Jr. J. Fusion of the left aortic cusp to the aortic wall with occlusion of the left coronary ostium, and aortic stenosis and insufficiency. Circulation, 1970;41:849–857.
2. Gibson R, Nihill M, Mullins C, Cooley D, Sandiford F, McNamara D. Congenital coronary artery obstruction associated with aortic valve anomalies in children: report of two cases. Circulation, 1981; 64:857–861.
3. Kurosawa H, Wagenaar S, Becker A. Sudden death in a youth, a case of quadricuspid aortic valve with isolation of origin of left coronary artery. Br Heart J, 1981;46:211–215.
4. Mutsuga M, Tamaki S, Yokoyama Y, Kato N, Yokote J, Ohata N, et al. Acute occlusion of left coronary ostium associated with congenital quadricuspid aortic valve. Ann Thorac Surg, 2005;79:1760–1761.
5. Oda T, Yasunga H, Todo K, Suda K. Case report – congenital repair of left coronary artery ostial isolation caused by aortic valve leaflet. Interactive cardiovascular and thoracic surgery, 2010; 11:796–797.
6. Shikata F, Nagashima M, Higaki T, Kawachi K. Congenital occlusion of the right coronary ostium by an aortic cusp attachment. Interactive cardiovascular and thoracic surgery, 2010;10:639–641.
7. Harada T, Fukae K, Ando Y. Surgical repair of ostial obstruction of the coronary artery for quadricuspid aortic valve in an infant. Interactive cardiovascular and thoracic surgery, 2017;24:634–635.
8. Samuel S, Shanantwari P, Meyer D. Myocardial ischaemia and valve insufficiency caused by a dysplastic aortic valve cusp: a previously unreported unique morphologic anomaly. Cardiology in the young, 2020; 30:1046–1049.
9. Gunsaulus M, Das N, Weinberg J, Kreutzer J, Medina M, DeBrunner M. Occlusion of the os of the left coronary artery by dysplastic aortic valve tissue presenting as progressive mitral insufficiency and cardiac arrest. Cardiology in the young, 2022; 1-3.
10. Tedga A, Pigula F, Lewis L, Kimball T. Quadricuspid aortic valve with guarded left coronary artery ostium in an infant with myocardial infarction. CASE, 2022; 7:6–9.
