# Supplementary figures and images for: Left coronary ostial isolation in a young boy caused by a dysplastic aortic valve: a case report
Source: Eur Heart J Case Rep. 2025 Jan 20;9(2):ytaf012. doi: 10.1093/ehjcr/ytaf012 (PMC11840340; doi:10.1093/ehjcr/ytaf012)

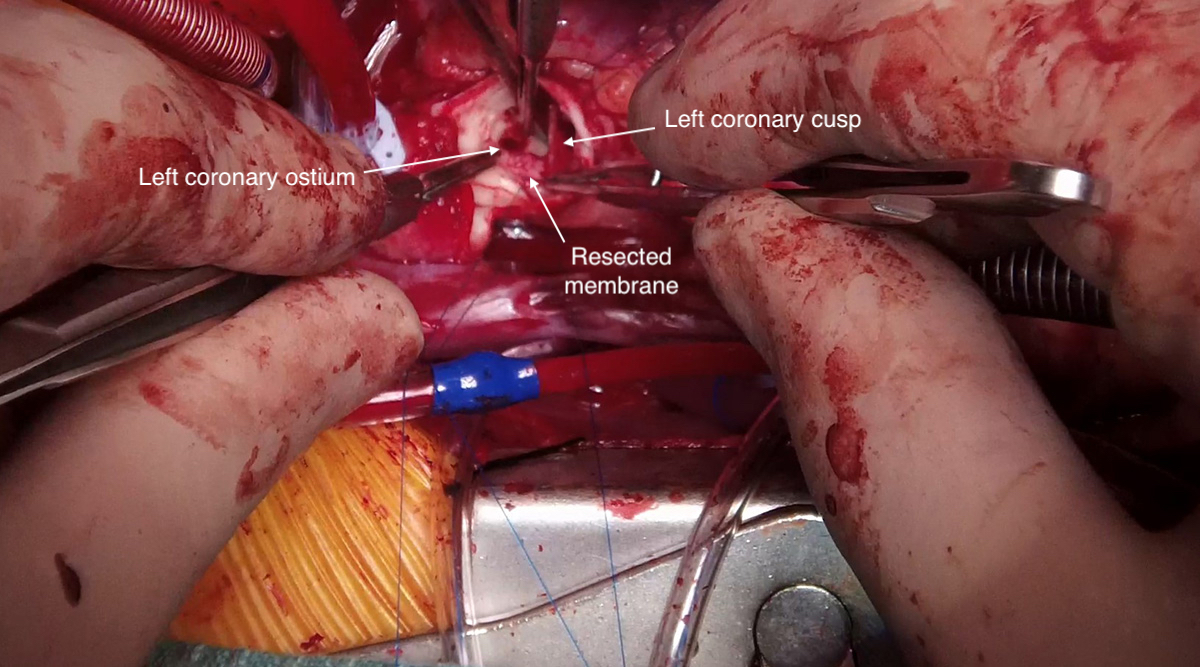

Supplement: ytaf012_Supplementary_Data [file ytaf012_supplementary_data.zip › Supp Figure 1B_Operative post membrane removal lab.jpg]

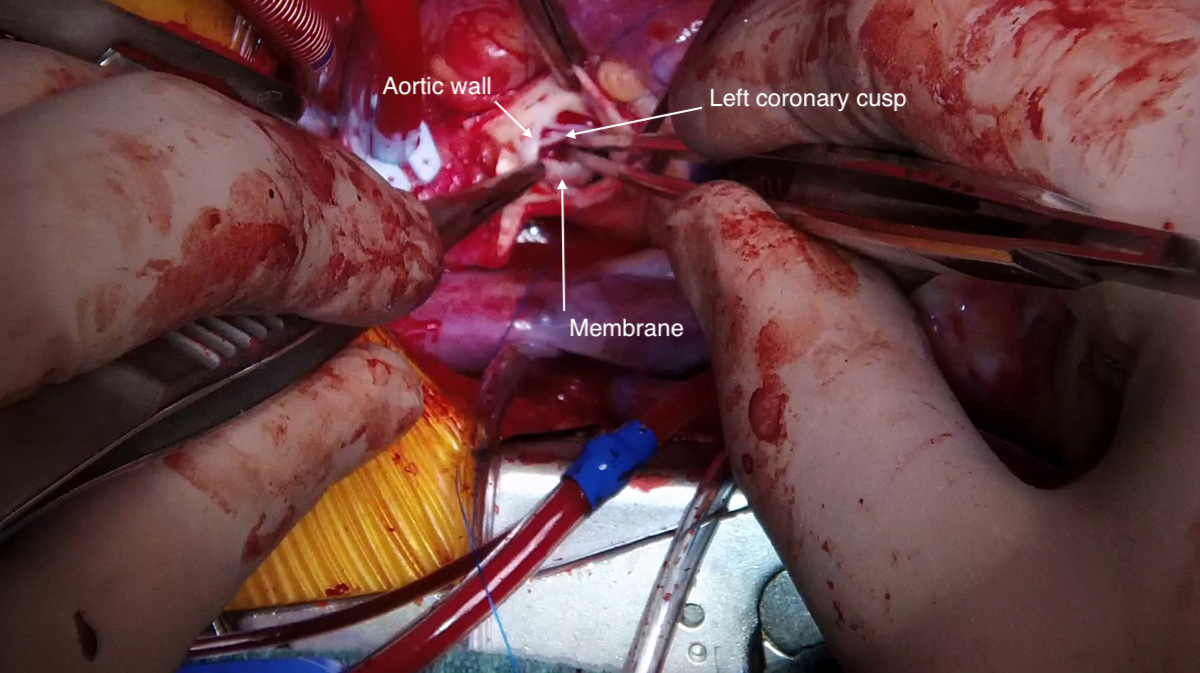

Supplement: ytaf012_Supplementary_Data [file ytaf012_supplementary_data.zip › Supp Figure 1A_Operative first look label.jpg]
